# 15 years of the model study course in medicine at the Ruhr University Bochum

**DOI:** 10.3205/zma001267

**Published:** 2019-10-15

**Authors:** A. Burger, B. Huenges, U. Köster, M. Thomas, B. Woestmann, H. Lieverscheidt, H. H. Rusche, T. Schäfer

**Affiliations:** 1 Ruhr University Bochum, Zentrum für Medizinische Lehre, Bochum, Germany; 2 Ruhr University Bochum, Abteilung für Allgemeinmedizin, Bochum, Germany

**Keywords:** problem-based learning, PBL, theme-oriented curriculum, integration, small group teaching

## Abstract

The Faculty of Medicine of the Ruhr University Bochum (RUB) introduced a model study course in medicine (MSM) in the winter semester 2003. For 9 consecutive years, 42 out of 280 first year students at the Ruhr University Bochum had the opportunity to begin their studies in the model study course in medicine. The places were allocated amongst the applicants internally through a raffle. The MSM was consistently problem-, practice- and patient-oriented and largely did away with lectures, broke with the distinction between a pre-clinical and clinical phase and tested basic knowledge in equivalent integrated exams focusing on clinical application. Following a comparative evaluation of the standard degree course (RSM) and the MSM, the faculty merged the two degree courses into the Integrated Reformed Medical Curriculum (IRMC), which has been on offer since 2013 and is characterized by a topic-oriented hybrid curriculum. This article examines experiences relating to the origins, conception and introduction of the MSM.

## Zusammenfassung

Zum Wintersemester 2003 führte die Medizinische Fakultät der Ruhr-Universität Bochum (RUB) den Modellstudiengang Medizin (MSM) ein. Für 9 aufeinanderfolgende Jahrgänge hatten je 42 von 280 StudienanfängerInnen der Ruhr-Universität Bochum die Gelegenheit, ihr Studium im Modellstudiengang Medizin zu beginnen. Die Plätze wurden unter freiwilligen Bewerbungen intern verlost. Der konsequent Problem-, Praxis- und Patientenorientierte MSM verzichtete weitgehend auf Vorlesungen, hob die Trennung von Vorklinik und Klinik auf und prüfte die Grundlagenkenntnisse in äquivalenten, integrierten Prüfungen mit klinischen Anwendungen. Nach vergleichender Auswertung von Regelstudiengang (RSM) und MSM führte die Fakultät die beiden Studiengänge zu dem seit 2013 angebotenen integrierten Reformstudiengang Medizin (iRM) zusammen, der durch ein themenorientiertes Hybridcurriculum gekennzeichnet ist. Dieser Artikel befasst sich mit den Erfahrungen mit der Entstehung, Konzeption und Einführung des Modellstudiengangs Medizin.

## 1. Foundation of the model study course

On October 18, 2001, the faculty council of the Faculty of Medicine of the Ruhr University Bochum decided to develop a model study course in Medicine (MSM) on the basis of the model study course clause of the new licensing regulations for physicians which were being revised at the time and began to develop the concept of patient-, practice- and problem-oriented teaching. 

The Office of the Dean of the Faculty of Medicine was the driving force behind this initiative. By boosting innovation through reforming medical studies, the faculty wanted to ensure its future of medical education in North Rhine-Westphalia. In 2001, the Office of the Dean appointed the head of the Department of General Medicine as the faculty representative for study reform. He assembled an interdisciplinary team with medical, pedagogical, psychological and social science background. Within two years, this “Office for Study Reform” (BfS) coordinated the development of the concept and the implementation of the model study course, which was offered to 42 students in parallel with the established standard medical degree course once the new licensing regulations came into force in the winter semester 2003/04. 

The underlying didactics were based on the principles of active learning in the sense of constructivism [1] with its four pillars of learner-centered active knowledge acquisition, linking to prior knowledge, learning support through social interaction, and application of learned content in authentic tasks [2]. Based on Harden’s SPICES model for curriculum development [3], the new study program’s teaching strategy was characterized by being student-centered, problem-oriented, the integration of basic and clinical training, the strengthening of General Practice; and a systematic learning spiral based on a catalog of learning objectives.

The curriculum was developed through exchanges with the medical faculties of Maastricht, Witten-Herdecke and Berlin, who already had experience with problem-oriented learning. BfS members and interested teaching staff participated in workshops in Berlin and Maastricht. The BfS was in close contact with the Office of the Dean of Studies of Medicine at the University of Witten-Herdecke. 

## 2. Organization and structural anchoring

The faculty tasked the Office for Study Reform (BfS) with managing the process, led by the faculty representative for study reform. 

The subjects listed in the Medical Licensure Act as relevant for medical education were asked to name one “coordinating departmental representatives” each who acted as a contact person in the following development process. For the cross-sectional areas, responsible people were designated by the Office of the Dean of Studies. The coordinating departmental representatives were tasked with collating the core learning objectives of their subjects and cross-sectional areas, from which the BfS compiled a learning content catalog. 

To develop the model study course, several working groups (WG) were formed [4] in which teaching staff, students and organizers were represented. 

The task of the **Reformed Degree Program WG** was to initiate and accompany the process. It laid the foundations for drafting the study and exam regulations [5]. 

**Figure 1:**
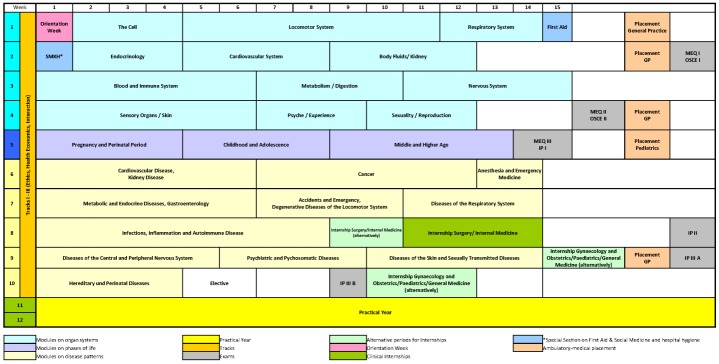
Theme-based curriculum of the medical model study program (horizontal: Semester weeks 1-16, vertical: Semester 1-12; MEQ/MCQ=combined modified essay question and multiple choice tests; OSCE=objective structured clinical examination)

The **Curriculum WG** had the task of restructuring the curriculum from the 1^st^ to the 10^th^ semester on the basis of the Bochum learning content catalog. The sequence of blocks (modules) and their contents were planned and defined. **Block Construction Groups** were formed for the individual blocks involving the respective departmental representatives. Based on a consensus template timetable, the block construction groups developed the final timetables by defining the content of each event and put the learning objectives of the thematic block in place through a defined and efficient systematic process [4]. Once the blocks content was established, **POL Case Construction Groups** were set up which, using real patients as a basis in order to fulfill the authenticity required for adult education [1], didactically prepared cases for use in POL groups according to module content of each week. These then formed the thematic framework of the respective semester week (see figure 1 (Fig. 1)).

At the start of the model study program an exam committee was formally set up as a public body to supervise the university internal equivalence exams replacing the 1^st^ State Examination and an evaluation commission for the prescribed evaluation of model study courses [5]. 

## 3. Changes in the departmental teaching/learning culture

The joint development of a new curriculum and the creation of an integrative learning content catalog made the relevance of interdisciplinary cooperation clear to everyone involved. Redundancies between the subjects were reviewed and an overall concept was developed that was transparent both for the teaching staff and the students [4]. 

In line with the current specific continuing education needs of the teaching staff and involving external expertise, medical didactic workshops were organized initially to qualify general medical teaching practices, curriculum development and problem-oriented learning. From this a comprehensive medical didactic continuing education curriculum, the Medical Didactics Bochum (MeDiBo), was developed, which today awards the Medical Didactics certificate nationwide, amounting to 120 hours of CPD [6]. 

All members of the teaching staff who were to lead a POL group with 7 students had to attend two days of tutor training. Here they learned how to support their POL groups in self-directed learning and how to provide feedback. This moved the issue of HOW to learn into focus and established a new learning culture. 

It was a new experience for the teaching staff that in POL students defined their own learning objectives and introduced them to each other, preferably in an interactive way. The objectives followed the learning interests of the students and could be consistent with the intended learning objectives but did not have to be. This self-directed learning initially led to uncertainty on both sides but was also very motivating. Over time, however, it became clear that this type of teaching and learning promoted communicative skills, dealing with one’s own uncertainties, self-directed learning, but also diagnostic skills [7]. Following Harden’s SPICES model, there was a deliberate change of perspective towards student self-responsibility with time reserved for self-study.

The joint commitment of the teaching staff and the students resulted in greater mutual appreciation and a different view of teaching. This manifests itself, amongst other things, in the way evaluation results are handled – tutors were graded and events which had received poor results were analyzed more closely. There was a “Teaching Newsletter”, in which innovative teaching concepts and evaluation results were presented. The performance-based allocation of funds (LOM), in which department heads receive funds depending on the course evaluations, also began and continues to reward good teaching.

Blended learning was also introduced systematically with the model study course, in which all events were accompanied by offers for pre- and post-work on a web-based learning platform (Blackboard). 

## 4. Contents

The contents of medical studies in Germany are clearly defined in the Medical Licensure Act (ÄApprO) [https://www.gesetze-im-internet.de/_appro_2002/BJNR240500002.html] and in the exam content catalog [8] published by the Institute for Medical and Pharmaceutical Exam Questions for the written parts of the state medical exam which faculties use as a guide in the sense of “constructive alignment” [9]. 

In § 1 of the study regulations for the MSM at the Ruhr University Bochum dated 20 April 2005, the faculty specified the reform objectives pursued in the model study course beyond the requirements of § 1 of the ÄApprO as called for in § 41 Sect. 2 No. 1 of the ÄApprO [5]: “Team communication and medical interaction skills, problem-oriented and interdisciplinary thinking as well as the readiness for lifelong learning should be promoted in particular.”

Criteria for the inclusion of illnesses into clinical teaching content were its frequency, the risk posed by it and its exemplary character. The teaching objectives were implemented in a consensual process between the subjects involved. To produce an integrated curriculum according to the so-called “Z model”, scientific basic knowledge was interlinked with clinical content from the first to the 10^th^ semester. Instead of systematic lectures, the relevant basic topics were suggested through carefully selected POL cases for independent work and studied in greater depth in accompanying seminars [10]. 

Three vertical tracks were developed to acquire overall competences:

- Medical interaction
- Health economics, scientific rigor, methodology and research
- Medical ethics, humanitarian aid, medical law and history of medicine

The contents of the three vertical educational tracks were systematically distributed throughout the course of study and were taken up again as part of a learning spiral in increasingly complex contexts. In doing so, early instruction in medical ethics proved conducive to maintaining a positive attitude towards ethical and medical-historical issues [11]. In the health economics, scientific, methodology and research track, students learned about the subject’s breadth from a basic understanding of the health care system and epidemiology to the development of their own research approaches. From the first semester on they learned how to handle statistical programs and developed and presented scientific questions.

All students were assigned to specially trained GP practices in which they completed five internships of 2 weeks each, distributed over the course of study. This allowed them to deepen and consolidate their knowledge, skills and abilities. The early and repeated contact with the GP practices strengthened the relationship with the patient and primary health care but also allowed the teaching doctors to observe the personal development of the students. General Practice was further strengthened by counting these internships as a clinical traineeship and through its integration into the various strands and numerous cross-sectional areas.

The students’ professional personal development was supported by POL tutors and mentors, with some of these links lasting beyond the period of university study. The BfS also performed important functions in study guidance and mentoring

## 5. Didactics

The curriculum largely dropped lectures as a format and relied on small group work (6 POL groups with 7 students each), which in turn were grouped in two seminar groups of 21 students each. The interdisciplinary lesson planning in thematic blocks followed a learning spiral based on the specially developed Bochum learning objective catalog. The first state examination was replaced by cumulative exams equivalent to the state examination (3 modified essay question tests and 2 OSCEs). 

The two weekly POL sessions of 1.5 hours each in semesters 1 to 5 and which consisted of self-directed learning using patient cases gave students the experience of being at the center of medical studies. This work in groups of 7 students was continued until the 10^th^ semester. Using a seven-step process [12] POL was implemented following Henk Schmidt as a method for knowledge acquisition [13] through problem discussion based on prior knowledge, recognition of knowledge gaps and how to close them through self-study and subsequent joint presentation of results and case-related discussion. 

**Figure 2:**
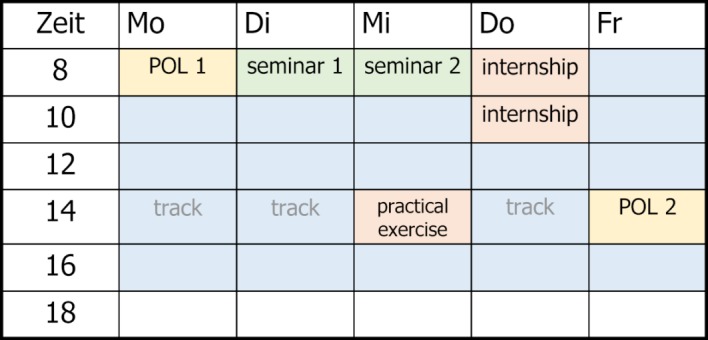
Template timetable for the first 2 years of study with 4 hours of POL, 4 hours of accompanying seminars, 4 hours of internships, 2 hours of practical exercises, 3 time slots for fortnightly events and time reserved for self-study (light blue).

Mandatory attendance was 18 teaching units per week during the first 5 semesters. The template timetable shows the relationship between POL group sessions, seminars, internships, exercises and reserved study times. Introduction to medical practice took place in the first semester by including clinical subjects and practicing medical skills through students examining each other. The first internship in a GP practice took place after the first semester, the others after the 3^rd^, 4^th^ and 9^th^ semesters. After the 5^th^ semester a two-week internship was scheduled in a specially trained pediatric practice (see figure 2 (Fig. 2)).

Exercises matching the block topics were held from the first semester as part of the internships, practical exercises and bedside teaching. In order to introduce students to different focus areas, a rotation took place between the large clinics of the University Hospital of the Ruhr University Bochum (UK-RUB).

The block logs, which were designed and maintained in the block construction groups, gave students an overview over the respective thematic block and hints for preparation and follow-up work, as well as broad learning objectives and literature recommendations. 

## 6. Examination/Evaluation

Interdisciplinary case-based exams equivalent to the first part of the state medical exam were introduced, adapted to the integrated theme-based curriculum. These consisted of a modified essay question test (MEQ) and an objective structured clinical examination (OSCE) with 7 stations, held after the first and second year of study. Exams on the remaining themes were held after the 5^th^ semester in a third MEQ. If all five exams were passed, this was counted as equivalent to the first state exam. As is the case with the state exam, each exam can be retaken twice. If a student failed to pass, they were permitted to continue studying until the 8^th^ semester. Exams on the clinical subjects and cross-sectional areas were held after the 5^th^ semester in combination with the MEQ3; after the 8^th^ and after the 10^th^ semester in case-related multiple-choice exams (MC). This change of method was justified on the one hand by higher reliability due to a wider spread of the exam contents and on the other hand to prepare for the second state examination, which is also an MC exam of a similar format. Interdisciplinary integration was achieved by asking questions from different subjects on the same case in order to be able to award subject-specific grades at the end.

**Figure 3:**
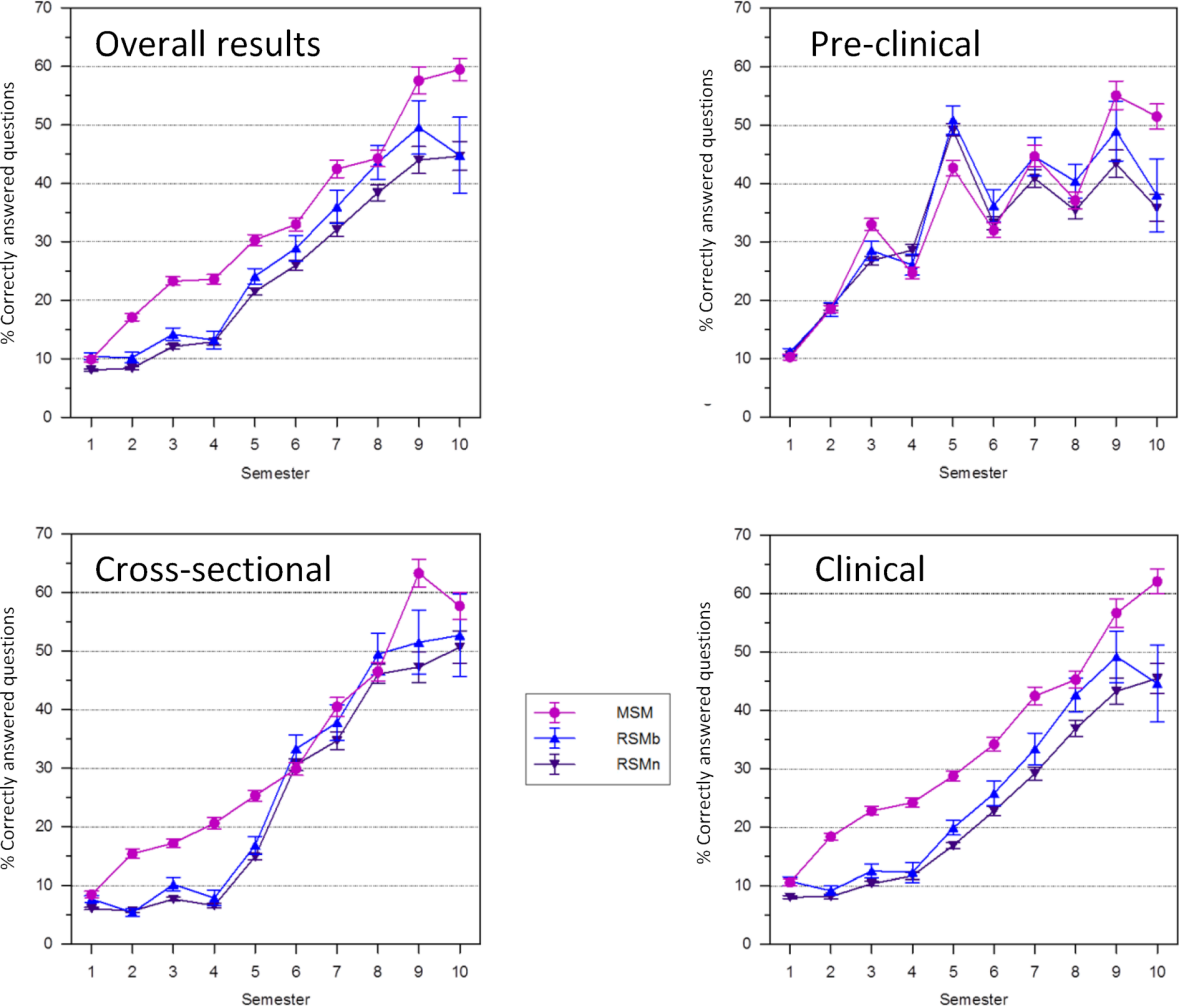
Summary of the first 10 progress test results from the winter semester 2003 to the summer semester 2008, divided into overall results of all questions, pre-clinical questions, questions on cross-sectional areas and clinical questions and separated into MSM students and RSM students who had unsuccessfully applied for the MSM (RSMb), and those who had not applied (RSMn).

The students’ knowledge acquisition in both degree courses was compared with the help of the formative Progress Test in Medicine (PTM) [14] developed at the Charité and the University of Witten-Herdecke. The MSM students achieved higher scores, which were attributed to better performance in questions concerning clinical practice (see figure 3 (Fig. 3)) [15]. The effect of the Z-curriculum which links basic and clinical content is visible from the first semester of study onwards in the MSM.

**Figure 4:**
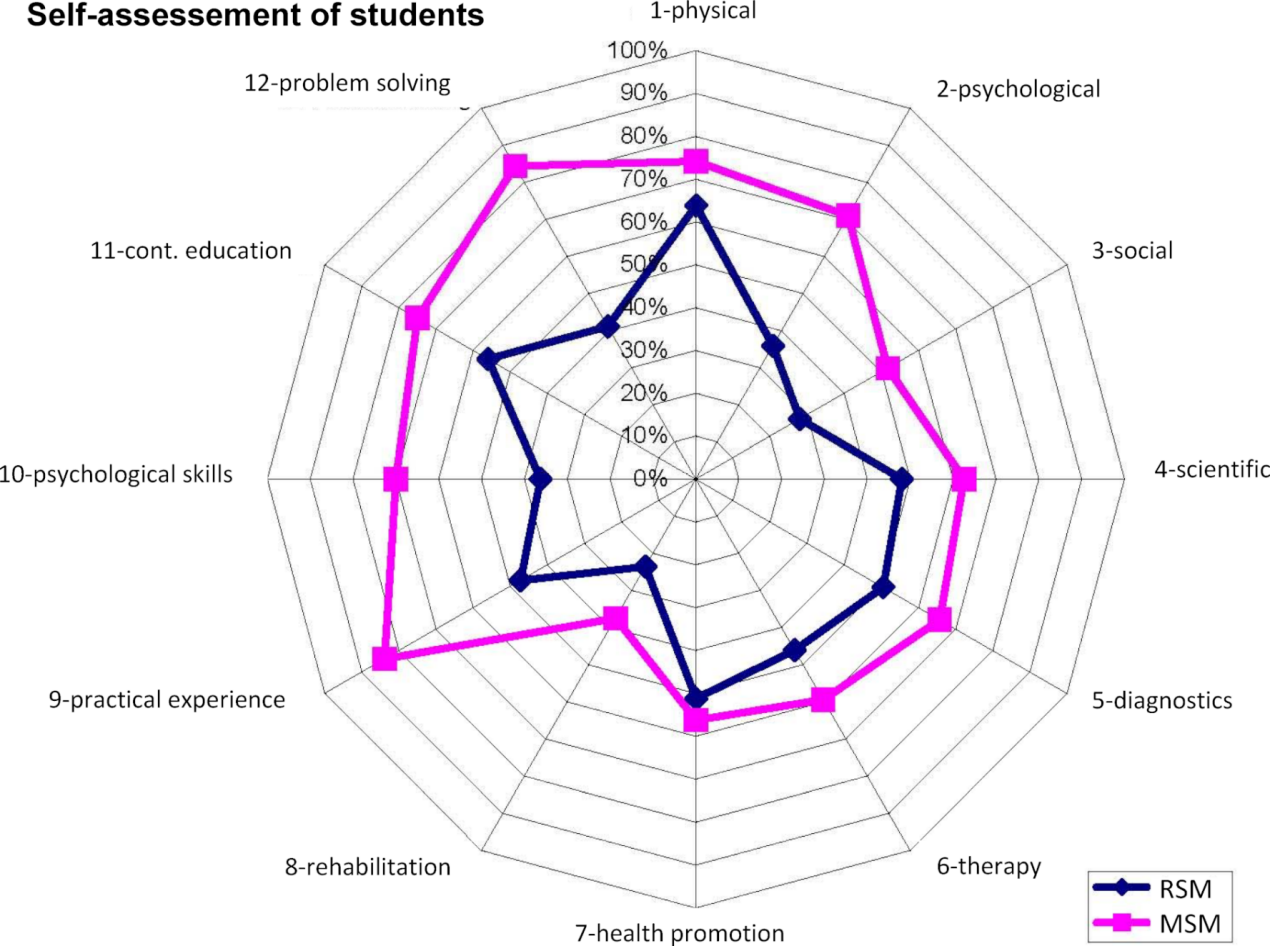
Results of self-assessment of students of the 10^th^ Semester with regard to the (full or almost full) achievement of the training objectives of the MSM as a percentage of participants (n=32/32 in the MSM, 127/187 in the RSM).

**Figure 5:**
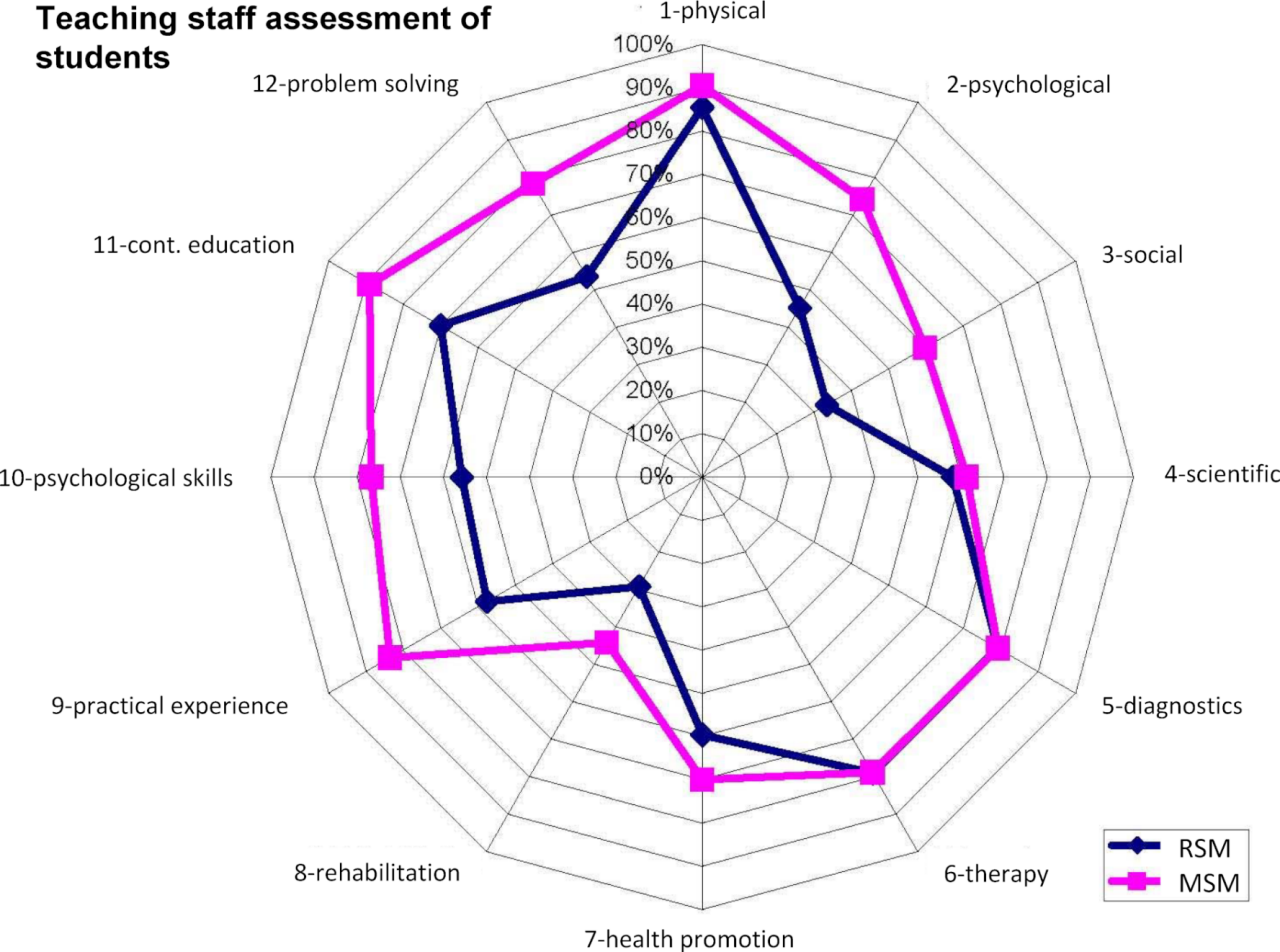
Results of teaching staff assessment of students of the 10^th^ Semester with regard to the (full or almost full) achievement of the training objectives of the model study course as a percentage of participants (n=85/375).

With regard to achieving the educational goals defined in the study regulations, both self- and third-party assessment ranked MSM students as more competent than those of the RSM (see figure 4 (Fig. 4)). Assessments by the teaching staff who taught in both programs confirmed this view (see figure 5 (Fig. 5)). However, in terms of diagnostic and therapeutic skills, scientific thinking and consideration of physical aspects of patients, both groups were estimated to be very close to each other. 

This was confirmed by the state exam results of the second part of the state medical exam, where a comparison of students from the two degree courses showed hardly any differences regarding success rates in answering specific questions [16].

Significant differences, however, were found with respect to the duration of studies. An analysis of the first three cohorts showed that 73.8%, 78.6% and 76.2% of the first year students passed the second state examination successfully and in the minimum period of study. In the standard degree course the respective rates were 51.9%, 51.3% and 49.2% 

All teaching courses and exams were assessed in writing. The evaluation results of all teaching courses were published regularly. In addition, at the end of each block there was a so-called block discussion between students, teachers and organizers. Beyond that, there were inter-block semester discussions with the spokespersons of the POL groups [17], [18].

## 7. Consolidation of the model and reformed standard degree course into the Integrated Revised Medical Curriculum (IRM)

After two cohorts had successfully completed the model degree course in 2011, the faculty initiated a new reform process to develop a new joint study program in medicine for the entire cohort of medical students enrolled at the Ruhr University. 

This reunification which had been a long-term goal was in particular demanded by the clinical stakeholders, as the large number of clinical events at the bedside (practical exercises with patients, examination courses, bedside teaching, elective blocks, practical year) for two different courses and with students with very different study progression constituted a significant logistical and content-related effort. 

Following extensive discussions and considerations of their own experiences and international experiences on integrated curricula [19], [20], [21], a newly established Curriculum WG under the direction of the Dean of Studies and supported by the ZML (which had emerged from the BfS in 2009) suggested the implementation of an Integrated Reformed Degree Program (IRDM) to the faculty council, involving teaching staff and students, which was to transfer aspects of the model study course in medicine to a much larger cohort: initially approximately 300 and later on 342 first year students. These included an integrated, theme-centered curriculum, early patient contact, strengthening of General Practice through GP practice placements in early semesters, continuing problem-oriented learning, organizing students in small fixed groups and establishing vertical training strands on medical interaction, hands-on medical skills, scientific rigor and the basics of medical thinking and acting. 

Simultaneous with the development of the IRDM, the medical scientific societies of the basic medical subjects expressed considerable reservations about model study courses in which the first state examination would be replaced by university internal exams. The first state examination, based on the exam content catalogs, was seen as a guarantor for the quality of medical education and perhaps also as a justification for the existence of the then basic subjects of the pre-clinical phase. The faculty council decided to apply to the state government for a model study course, retaining the first state examination and with a possibility of doing the clinical traineeship at a different time so that the GP practice placement program could begin during the first two years of study; and for these placements to count as a clinical traineeship as in the case in the MSM. The Ministry of Health turned down this request. As a result the faculty decided to formally implement the IRDM as their standard degree course. 

The development of the new IRDM curriculum was coordinated by the ZML and the Office of the Dean of Studies and was developed at regular conferences of the departmental representatives of the subjects involved in the respective phase of studies and with the participation of student representatives. The experiences from the development of the MSM were very helpful in this undertaking.

In contrast to the MSM, the POL groups were increased from 7 to 10 participants and the frequency of the POL sessions was reduced, leading to the POL cases taking on a different function in the curriculum. They no longer encompass the entirety of knowledge to be acquired in self-study and accompanying seminars but rather challenge students to transfer knowledge acquired in thematic blocks to clinical cases. For this purpose, new template timetables were developed, which provide a systematic introduction to the topic through lectures, followed by seminars and, finally, internships to apply learned content, flanked by the POL sessions, which take place in 34 groups of 10 students each.

A three-day shadowing internship in a GP surgery after the third semester is systematically prepared right from the start through a total of 100 teaching hours where, using the “Z Model”, clinical content is linked with basic content during the pre-clinical phase as part of the practical introduction to clinical medicine and medical careers. 

Based on the National Competence-Based Catalog of Learning Objectives (NKLM), the medical interaction track and the scientific track have been expanded and anchored vertically in the curriculum. 

The first cohort of the IRDM will graduate in autumn 2019. 

## Conclusion

The model study course in medicine at the Ruhr University emphasized problem-, patient- and practice-orientation of studies. It used problem-oriented learning as a structured form of teaching in a theme-based, interdisciplinary curriculum, flanked by seminars, consultations, internships and practical exercises, which right from the first semester onwards were jointly taught by lecturers from the pre-clinical, clinical and theoretical phases. By largely dropping systematic lectures, students were given sufficient time for self-study. As part of the GP shadowing program, students received long-term mandatory exposure to General Practice. The results of the progress test in medicine and the 2^nd^ part of the state examination showed that students did not lag behind in knowledge acquisition and that the targeted learning objectives, amongst other, in problem solving competence, practical experience and scientific rigor were rated higher by students and the teaching staff of the model study course. 

The development and implementation of the model study course in parallel to a (revised) standard course awarded teaching a much higher importance than before at the faculty. It led to the establishment of a medical didactic qualification program, resulted in experiences with new teaching and examination formats, developed a culture of joint exchange and evaluation of teaching-related content and paved the way for a targeted development of a new integrated reformed degree program, which implements a Z-curriculum that interlinks pre-clinical and clinical content as part of the structure prescribed by Medical Licensure Act for standard degree courses. 

## Competing interests

The authors declare that they have no competing interests.
